# DC-Based Immunotherapy Combined with Low-Dose Methotrexate Effective in the Treatment of Advanced CIA in Mice

**DOI:** 10.1155/2015/834085

**Published:** 2015-06-28

**Authors:** Jun-Eui Park, Jinah Jang, Ji-Hye Choi, Mi-sun Kang, Yun-Ju Woo, Young-Rim Seong, Chan-Bum Choi, Hye-Soon Lee, Sang-Cheol Bae, Yong-Soo Bae

**Affiliations:** ^1^JW CreaGene Research Institute, JW CreaGene Inc., Seongnam-si, Gyeonggi-do 462-120, Republic of Korea; ^2^Department of Rheumatology, Hanyang University Hospital for Rheumatic Diseases, Seoul 133-791, Republic of Korea; ^3^Department of Biological Science, Sungkyunkwan University, Suwon, Gyeonggi-do 440-746, Republic of Korea

## Abstract

We have previously demonstrated that semimature dendritic cell- (smDC-) based immunotherapy is effective for the treatment of collagen-induced arthritis (CIA) prior to disease onset. In the present study, we examined the efficacy of combination therapy with smDCs and methotrexate (MTX) in advanced CIA with a score of 2-3. Combination therapy with low-dose MTX and type II collagen- (CII-) pulsed smDCs (CII-smDCs) was more effective in inhibiting disease progression than high or low-dose MTX alone or a combination of high dose MTX and CII-smDCs. The effect of CII-smDCs alone was also comparable to the combination therapy. CD4^+^Foxp3^+^ Treg populations and IL-10 secretion markedly increased, and CII-specific autoreactive T cells decreased in mice treated with CII-smDCs alone or in combination with MTX. Combination therapy reduced the secretion of interferon-*γ* (IFN-*γ*) and IL-17 with little influence on the IL-4 secretion in the mixed leukocyte reaction. These results imply that the combination therapy with low-dose MTX and smDCs is effective in controlling advanced CIA by enhancing Treg population and suppresses antigen-specific Th1/Th17 immunity, rather than initiating Th1 to Th2 immune deviation. Our findings provide a better understanding of the DC therapy in combination with MTX for the treatment of patients with rheumatoid arthritis (RA).

## 1. Introduction

RA is an autoimmune disorder characterized by chronic inflammation of the synovial joints leading to destruction of cartilage, bone, and ligament. The conventional treatment of RA with disease modifying antirheumatic drugs (DMARDs) aims to alleviate disease symptoms and delay or prevent future joint destruction. Advanced therapies are targeting inflammatory cytokines and autoreactive T cells, since the imbalance of inflammatory/anti-inflammatory cytokines is an important factor in RA pathogenesis [1].

Dendritic cells (DC) are professional antigen-presenting cells (APC) and have an important role in both innate and adaptive immunity [2]. They are crucial for mediating immune system activation via antigen uptake and presentation to T cells. Dendritic cells also mediate immune tolerance to self-antigens and prevent excessive immune response [3–5]. The basic knowledge surrounding tolerance induction has been greatly strengthened by the studies of several groups that generated human tolerogenic DCs (tolDCs)* in vitro* [6–8]. TolDCs contribute to tolerance restoration through the induction of activation-induced cell death, anergy, and/or regulatory T cells in autoimmune diseases [9, 10]. A clinical trial that used tolDCs to treat type I diabetes showed no severe adverse effects and opened the possibility of tolDC use to treat autoimmune disease [11]. Although it remains unclear whether DCs have a role in the initiation of RA pathogenesis, evidence points toward a significant role for DCs in the maintenance and progression of RA [9]. Nevertheless, emerging therapies for RA are exploiting the tolerogenic capacity of DCs [9]. There are several clinical trials in progress that have exhibited that DC therapy is safe for the treatment of RA [12, 13].

We previously reported that semimature DCs (smDCs) have shown tolerogenic potential and a preventive effect when inoculated at a low dose in mice with collagen-induced arthritis (CIA) [14]. However, the effect of smDCs or other tolDCs has never been demonstrated in an advanced RA model. In the present study, we evaluate the therapeutic effect of smDCs in combination with methotrexate (MTX) in advanced CIA mice.

MTX is a first line DMARD in most RA patients since MTX has a great efficacy/toxicity ratio [15]. It is a typical folic acid antagonist and also has an antirheumatic effect by antiproliferative, anti-inflammatory, and immunosuppressive mechanisms [16]. It improves clinical symptoms and slows joint damage. MTX itself has immunosuppressive and anti-inflammatory properties [17]. It has been reported that MTX treatment inhibits TNF-*α* production and correlates with prevention of disease progression in a T cell-dependent mouse CIA model [18, 19]. A possible mechanism is partial induction of regulatory T cells (Treg), induction of Th1-to-Th2 shift, and downregulation of Th1 cytokines [16, 20]. However, the effect of MTX in an advanced CIA model has never been reported.

In the present study, we show the therapeutic effect of smDCs in combination with MTX in an advanced CIA model. Here, we found that combination therapy with smDCs and low-dose MTX was effective in controlling disease progression in advanced CIA mice. The benefit was likely associated with reduction of antigen-specific Th1 and Th17 populations in the spleen and an increase in the LN Treg population after treatment. There were no adverse effects using the combination of smDC and MTX. This data will help us in our pursuit of using smDC in the treatment of RA, either with or without MTX.

## 2. Materials and Methods

### 2.1. Mice and Reagents

Six- to eight-week-old pathogen-free female DBA/1J mice were purchased from Charles River Japan (Atsugi, Kanagawa, Japan) and maintained in the Animal Facility of the JW CreaGene Research Institute (Gyeonggi, Republic of Korea). All experiments were conducted in accordance with local animal ethics guidelines and approved by the Institutional Animal Care and Use Committee (IACUC).

Cells were cultured in RPMI 1640 (Gibco Laboratories, Grand Island, NY, USA) supplemented with 10% fetal bovine serum, 50 nM 2-mercaptoethanol (Life Technologies, Gaithersburg, MD, USA), 100 *μ*g/mL streptomycin, and 100 U/mL penicillin. Recombinant murine granulocyte-macrophage colony-stimulating factor (GM-CSF) and recombinant murine interleukin 4 (IL-4) were obtained from JW CreaGene. Complete Freund's adjuvant (CFA), incomplete Freund's adjuvant (IFA), collagen (type II collagen, chicken), LPS, and MTX were obtained from Sigma-Aldrich (St. Louis, MO, USA). CFSE (5,6-carboxyfluorescein succinimidyl ester) was obtained from Invitrogen (Waltham, MA, USA). Recombinant mouse tumor necrosis factor *α* (TNF-*α*) and CCL19 were purchased from R&D Systems (Minneapolis, MN, USA). PE-conjugated anti-mouse MHC II, CD40, CD11c, CCR7, and CD80 and FITC-conjugated anti-mouse CD86, CD54, and CD14 were purchased from BD Pharmingen (San Diego, CA, USA). PE-conjugated anti-mouse Foxp3 was purchased from eBioscience (San Diego, CA, USA).

### 2.2. Generation of Mouse Bone Marrow-Derived smDCs

Dendritic cells were generated from bone marrow progenitors of mice as described previously with some modifications [14]. Briefly, bone marrow cells were prepared from femurs and tibias of mice, and RBCs were lysed with ACK lysing buffer (CMABREX Bio Science, Walkersville, MD, USA). Cell suspensions were cultured in bacterial plates in the presence of 20 ng/mL of GM-CSF and 2 ng/mL of IL-4. At day 3, nonadherent cells were washed and refed in the same culture conditions. After 10 days of culture, the semimature and mature DCs were generated by additional incubation with 500 U/mL TNF-*α* and 50 *μ*g/mL type II collagen (CII) for 4 h and with 1 *μ*g/mL of LPS and 50 *μ*g/mL CII for 24 h, respectively.

### 2.3. Flow Cytometry Analysis

Direct immunofluorescence staining was performed as described previously to analyze the DC surface phenotypes [21]. Dendritic cells were stained with appropriate antibodies at 4°C for 20 min. After washing, cells were analyzed by FACS Caliber (BD) using CellQuest or FlowJo software.

### 2.4. Chemotaxis of smDCs

To test DC migration* in vitro*, the lower chambers of a transwell system (8 *μ*m pore size, 24 well plate, Corning, NY, USA) were filled with CCL19 (Mip3-*β*, 300 ng/mL) in 0.6 mL and DCs (1 × 10^5^ cells in 0.1 mL) in serum-free RPMI1640 medium were placed in the upper chambers. Plates were incubated for 3 h at 37°C in 5% CO_2_ to allow for migration. Migrated DCs were harvested from the lower chambers, and the total number of cells was determined by cell counting.

### 2.5. Induction of Collagen Induced Arthritis (CIA) and Treatment

CIA was induced as reported previously [14]. DBA/1J mice were immunized subcutaneously with 200 *μ*g of type II collagen (CII, 2 mg/mL) dissolved in 100 *μ*L of 0.05 M acetic acid and mixed with an equal volume (100 *μ*L) of CFA. On day 21, mice were boosted with a subcutaneous injection of CII mixed in IFA. CIA mice were treated with MTX (0.1 mg/kg, 0.5 mg/kg, or 1.0 mg/kg), vaccinated with CII-pulsed smDC (2 × 10^5^ cells/mouse), and/or treated by combination therapy with CII-smDC + MTX (0.1, 0.5, and 1.0 mg/kg). CIA mice with an arthritic score of 2-3 (5-6 weeks after primary CII-inoculation) were treated with two consecutive cycles of MTX intraperitoneally three times a week starting on day 39 and with CII-smDCs injected subcutaneously once a week starting on day 44. Each therapeutic cycle was completed by administration of MTX on days 2, 4, and 6, followed by administration of CII-smDCs on day 7. Animals were sacrificed on day 77 for histological studies and immune status evaluation. Additionally, cells from spleen and draining lymph nodes were cultured for 72 h in the presence of 50 *μ*g/mL of CII and the Treg population and IFN-*γ*/IL-4/IL-17/IL-10/TGF-*β* secretion from the culture supernatant was determined via FACS analysis and ELISA.

### 2.6. Evaluation of Arthritis

The degree of arthritis was evaluated from 28 days after the first immunization to up to 8 weeks. The severity of arthritis was expressed as a mean arthritis index on a 0–3 scale, as follows: 0: normal joint; 1: slight inflammation and redness; 2: severe erythema and swelling affecting the entire paw, with inhibition of use; and 3: deformed paw or joint, with ankylosis, joint rigidity, and loss of function. The total score for clinical disease activity was based on all four paws, with a maximum score of 12 for each mouse. Scoring was done by two independent observers, without knowledge of experimental protocols. Footpad thickness was measured twice a week using a caliper.

### 2.7. Histopathology

After sacrificing the mice, knee joints were dissected, fixed in 10% phosphate-buffered formalin for 2 days, decalcified in 10% EDTA for 7 days, dehydrated in an alcohol gradient, and rinsed in running water. The specimens were processed for paraffin embedding in Paraplast (BDH, Dorset, UK) as routine procedure. Serial paraffin sections measuring 5 *μ*m were cut along a longitudinal axis throughout the joint on a microtome, heated at 60°C for 30 min, and deparaffinized. Hydration was done by transferring the sections through the following solutions: 70% ethanol, 80% ethanol, 95% ethanol, and 100% ethanol for 2 min twice and xylene for 6 min three times for clarity. The sections were stained with Hematoxylin and Eosin (H&E) and mounted on glass slides.

### 2.8. T Cell Proliferation Assay

T cell proliferation assays were performed as described previously [22]. Splenocytes were isolated and T cells were purified using nylon wool. Splenic T cells were resuspended in PBS (0.1% BSA) at 1 × 10^6^ cells/mL and incubated with 1 *μ*M CFSE (Invitrogen) for 30 min. DCs (1 × 10^4^) were cultured with CFSE-labeled spleen T cells (1 × 10^5^) for 5 days in 96-well round-bottomed plates (Corning, NY, USA). After 5 days, cells were harvested and the CFSE signal of gated lymphocytes was analyzed by flow cytometry.

### 2.9. Cytokine Analysis

Spleen and lymph nodes were isolated from mice at the peak of clinical CIA (on day 77). After removal of red blood cells by treatment with 0.16 M Tris-NH_4_Cl solution, spleen cell suspensions (1 × 10^6^ cells/mL) were incubated at 37°C for 72 h in complete RPMI 1640 medium containing 50 *μ*g/mL CII. After 72 h incubation, the supernatants were collected and IFN-*γ*, IL-4, IL-17, IL-10, and TGF-*β* levels were measured using commercially available ELISA kits (Quantikine Mouse ELISA kit, R&D Systems, Minneapolis, MN, USA) according to the manufacturer's protocol.

### 2.10. CD4^+^Foxp3^+^ Treg Cells

To determine CII-induced CD4^+^Foxp3^+^ Treg cells, spleen and LN cells were first stained with FITC-conjugated anti-CD4 antibodies and then fixed and permeabilized with the BD Cytofix/Cytoperm kit (BD Bioscience Pharmingen). Cells were then stained with PE-conjugated anti-mouse Foxp3 antibody in BD perm/wash buffer for 1 h. After washing with BD perm/wash buffer, cells were analyzed by flow cytometry, in which CD4^+^Foxp3^+^ cells were gated from live lymphoid cells.

### 2.11. CII-Specific T Cell Response* In Vitro*

*In vitro* proliferation of splenocytes was examined using a 3-[4,5-dimethylthiazol-2-yl]-2,5 diphenyltetrazolium bromide (MTT) assay. On day 77, splenocytes (1 × 10^5^ cells/well in a round bottom, 96-well plate) were cultured for 5 days at 37°C in RPMI medium containing 50 *μ*g/mL CII. On the day of the assay, MTT (0.5 mg/mL) was added to the medium in each well, and plates were returned to the incubator for 4 hr. Supernatants were removed and 100 *μ*L of dimethyl sulphoxide was added. The absorbance of the samples was then recorded at 570 nm with background subtracted at 630 nm. Data are presented as the percentage of the cells cultured with medium alone.

### 2.12. Measurement of CII-Specific Antibodies in the Serum

The levels of anti-CII antibodies from serum were determined by enzyme-linked immunosorbent assays (ELISAs) as described previously [23]. Briefly, ELISA plates (Nunc-Immunos, Denmark) were coated with 4 *μ*g/mL of CII in 50 mM carbonate buffer at 4°C overnight. Fifty microliters of mouse sera diluted at 1/2,000 was added to each well and incubated at RT for 1 hr. Subsequently, the plates were incubated with alkaline phosphatase conjugated goat anti-mouse IgG (Southern Biotechnology Associates, Birmingham, AL, USA) and then washed and developed for 5–20 minutes in a dark room by the addition of* p*-nitrophenyl phosphate (Sigma). Antibody titers are expressed as OD_405_ units.

### 2.13. Quantitative Real-Time PCR

qRT-PCR was performed as described previously [22]. Total RNA was extracted with Trizol (Invitrogen Life Technologies). cDNA synthesis was performed with the Stratascript II reverse transcriptase (Invitrogen Life Technologies). PCR was performed using a SYBR based assay to examine the level of Foxp3 and cytokine mRNA with Power SYBR Green PCR Master Mix (Applied Biosystems, Carlsbad, CA, USA) on the StepOnePlus Real-Time PCR System (Applied Biosystems). The PCR primers are shown in Table 1. Samples were in duplicate and relative expression was determined by normalizing to *β*-actin expression in each set of samples to calculate a fold-change value.

### 2.14. Coculture of Arthritogenic T Cells with smDCs and MTX

Arthritogenic T cells collected from the spleen of CIA mice at day 21 from first CII injection were cocultured with CII-pulsed smDC at a 10 : 1 ratio for 6 days in the absence or presence of MTX from 0 to 5000 nM. Cells were first stained with FITC-conjugated anti-CD4 antibodies and then fixed and permeabilized with the BD Cytofix/Cytoperm kit (BD Bioscience Pharmingen). Intracellular IFN-*γ* and Foxp3 were then assessed after staining with PE-conjugated anti-mouse IFN-*γ* antibody or PE-conjugated anti-mouse Foxp3 antibody, respectively, in BD perm/wash buffer for 1 h. After washing with BD perm/wash buffer, CD4^+^IFN-*γ* ^+^ T cells or CD4^+^Foxp3^+^ T cells were analyzed by flow cytometry from a live lymphocyte gate.

### 2.15. Statistical Analysis

Student's *t*-test was used for all statistical analyses. A *p* value of less than 0.05 was considered statistically significant.

## 3. Results

### 3.1. smDCs Have Tolerogenic Potential in* In Vitro* Immunological Analysis

**Figure 1:**
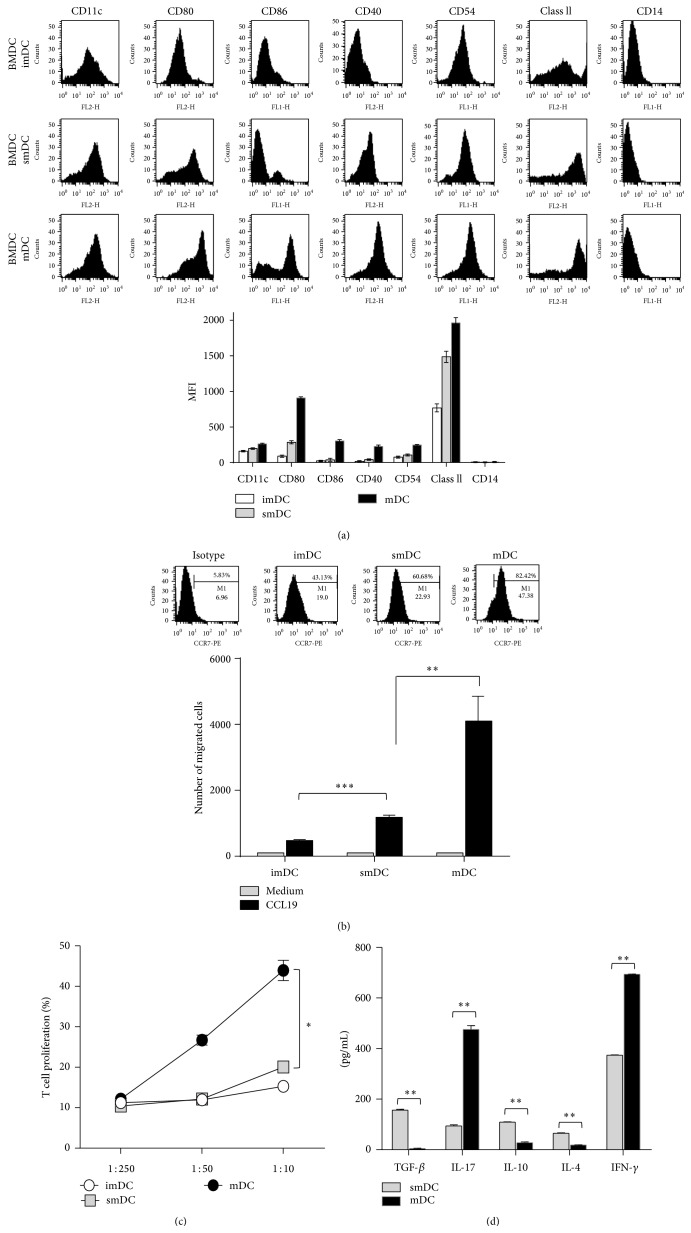
Surface phenotypes and characteristics of smDCs. (a) Representative FACS data showing the surface phenotype of bone marrow cell-derived imDCs, smDCs, and mDCs (upper), and geometric mean fluorescence intensities (gMFI) of each surface molecule are shown (lower). (b) The expression level of CCR7 on the surface of imDCs, smDCs, and mDCs was assessed by FACS after staining with anti-CCR7 mAb. CCR7^+^ cell populations in each representative FACS data are indicated (upper). The migration of imDCs, smDCs, and mDCs was assessed by an* in vitro* CCL19/MIP-3*β* chemotaxis assay as described in Section 2. DCs were seeded in the upper chamber, and the number of migrated DCs was analyzed after 3 h. (lower). (c) T cell stimulation capacity of each DC subset. Purified CFSE-labeled splenic T cells were mixed with each syngeneic imDCs, smDCs, and mDCs at different ratios for 5 days. (d) Cytokine production was determined in primary cultures at DC/T cell ratios of 1 : 10. The supernatants were collected for IFN-*γ*, IL-10, IL-17, IL-4, and TGF-*β* production as determined by ELISA. The statistical data in (b)~(d) are shown as mean ± SEM from more than 3 independent experiments. ^*∗*^ *p* < 0.05, ^*∗∗*^ *p* < 0.01, ^*∗∗∗*^ *p* < 0.001 in Student's *t*-test.

TNF-*α* modulated smDCs revealed the semimature phenotypes as shown previously [14], that is, high levels of expression of MHC II and lower levels of expression of costimulatory molecules such as CD80, CD86, and CD40 compared with mDC (Figure 1(a)). The expression of CCR7 was relatively low in smDCs and increased by maturation (Figure 1(b) upper). The transwell experiment showed that the Mip3*β*-dependent migration capacity of smDCs was similar to the pattern of CCR7 expression (Figure 1(b) lower). smDCs have much lower T cell proliferation capacity than mDCs in coculture experiments with splenic T cells (Figure 1(c)). We examined the cytokines produced from T cell/DCs coculture in culture supernatant using ELISA. Different from mDCs, smDCs produced relatively high anti-inflammatory cytokines (immunosuppressive cytokines), such as TGF-*β*, IL-4, and IL-10, rather than proinflammatory cytokines such as IL-17 and IFN-*γ* (Figure 1(d)). These results suggest that smDCs would be involved in immune regulation rather than immune system activation. We also found that Treg cell population increased when smDCs were incubated with mouse splenocytes (data not shown).

### 3.2. Combination Therapy with Low-Dose MTX and smDCs or smDC Vaccine Alone Ameliorated Advanced Arthritis

**Figure 2:**
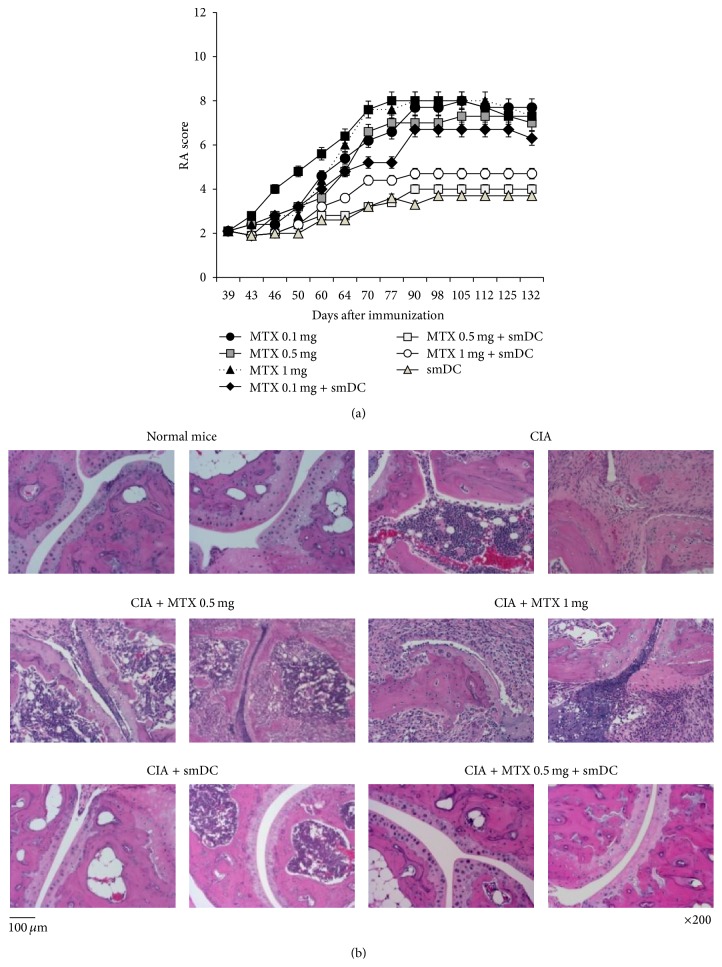
Treatment of advanced CIA mice with smDCs alone or in combination with MTX. (a) DBA/1J mice were injected with CFA-mixed type II collagen (CII) on days 0 and 21. Advanced CIA mice (RA score 2~3) were then treated with smDC and/or MTX for 2 weeks. Disease progression was monitored for 132 days. (b) Histological analysis of the hind paws. Hind paws were removed from mice in all groups at day 77 after first CII injection and fixed in paraffin blocks. Histopathology was examined after H&E staining as described in Section 2.

We showed that lower doses of CII-pulsed smDCs (2 × 10^5^) can prevent CIA when treated before disease onset [14]. In the present study, we examined the therapeutic effect of CII-pulsed smDCs in advanced CIA mice in combination with MTX since most of RA patients are normally treated with MTX until showing MTX-resistance. When the disease activity score of CIA mice reached 2~3, three different doses of MTX (oral route, 3 times/week) and/or smDCs (subcutaneously, once/week) were treated for 2 weeks and the RA score was examined. In order to determine the optimal dose of MTX for combination therapy with smDCs, different doses (from 0.1 mg/kg to 1 mg/kg) of MTX were tested in combination with or without smDCs. When mice were treated with smDCs and 0.5 mg/kg or 1 mg/kg MTX, disease progression was significantly delayed. Treatment with MTX alone did not result in any significant difference in disease progression (Figure 2(a)). In other words, the severity of arthritis was effectively controlled within the range of arthritic score 3~4 by smDCs alone or by combination therapy for longer than 132 days in CIA mice, while the untreated control group reached scores of 7~8 on day 70 and maintained these scores up to day 132 (Figure 2(a)).

Joint histopathology data supports the preclinical arthritis score. As shown in the representative H&E staining, both untreated control CIA mice and CIA mice treated with MTX alone showed significant joint infiltration by leukocytes and severe disruption and loss of articular cartilage (Figure 2(b)). The CIA group treated with smDCs alone or in combination with MTX (0.5 mg/kg) showed nearly intact articular cartilage and subchondral bone and less synovial hyperplasia in the histopathologic analysis (Figure 2(b)).

Interestingly, treatment with smDCs alone was also as effective as combination therapy with 0.5 mg/kg MTX in controlling disease progression of CIA mice, but the combination of 0.1 mg/kg MTX + smDCs did not show any therapeutic effect (Figures 2(a) and 2(b)). These data suggest that RA patients taking MTX can be treated with smDC vaccines without disturbing MTX treatment because MTX is a standard therapy for early RA patients.

### 3.3. Antiarthritic Effects of smDCs or Combination Therapy with MTX Were Associated with Long-Term Immune Hyporesponsiveness

**Figure 3:**
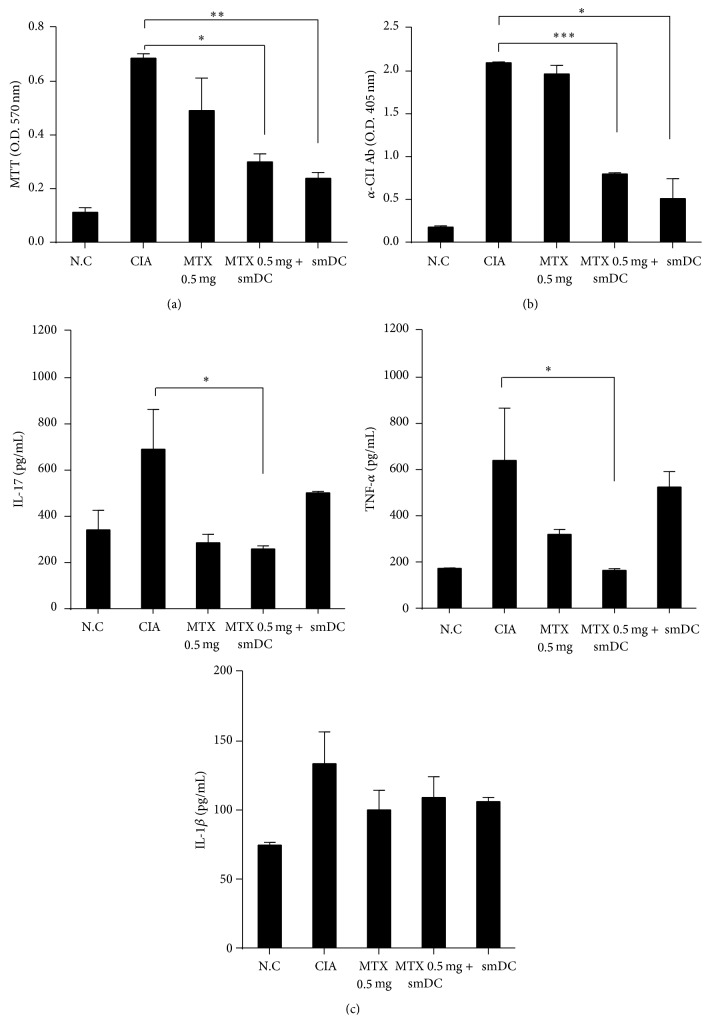
Analysis of CII-specific splenocytes and the levels of CII-specific IgG and proinflammatory cytokines in CIA mice after a combination therapy. (a) Advanced CIA mice were treated with smDCs, MTX, or smDCs and MTX. Splenocytes were isolated from mice on day 77 after first CII injection and stimulated by culturing in the presence of CII (50 *μ*g/mL) for 72 h. Cell proliferation was measured by an MTT assay. (b) Serum was collected from each mouse on day 77 after first CII injection. Anti-CII antibody (Ab) levels were measured by ELISA. (c) Serum samples were collected from normal controls and CIA mice at 77 days after first CII injection and anti-IL-17, TNF-*α*, IL-1*β* antibody (Ab) levels from serum were measured by ELISA. Statistical data are shown as mean ± SEM from more than 3 different sets of experiments. ^*∗*^ *p* < 0.05, ^*∗∗*^ *p* < 0.001, ^*∗∗∗*^ *p* < 0.0001 in Student's *t*-test.

Since it was reported that T cells play an important role in CIA onset [24] and smDCs can induce immune tolerance by T cell hyporesponsiveness [25], we evaluated CII-specific T cells by proliferation assay with splenic T cells from each group of mice. In comparison with an untreated control group of CIA mice, CII-specific T cell proliferation was significantly decreased in mice treated with smDCs alone or in combination with 0.5 mg/kg MTX (Figure 3(a)). These data suggest that the preclinical implications shown in Figure 2 would be associated with CII-specific T cell hyporesponsiveness after the treatment of CIA mice with smDCs alone or in combination with MTX. Treatment with MTX (0.5 mg/kg) alone also reduced CII-specific T cell proliferation to some amount in CIA mice (Figure 3(a)), suggesting that the hyporesponsiveness induced by MTX (0.5 mg/kg) alone would not be enough to control disease progression.

CIA mouse serum contains profound amounts of CII-antibody at the time of disease onset [26]. It has also been suggested that tolDCs are involved in the inhibition of B cell-mediated antibody production by blocking T-helper cell function [27].* In vitro* generated smDCs prevent arthritis and inhibit anti-CII antibody production in CIA mice [25, 28]. We examined the level of anti-CII antibody in CIA mouse serum after treatment with smDCs. Forty days after the onset of RA, anti-CII antibody levels decreased significantly in mice treated with smDCs alone or in combination with MTX, while treatment with MTX alone resulted in no change in serum antibody level (Figure 3(b)), suggesting that there would be a correlation between preclinical outcome and anti-CII antibody level.

Levels of proinflammatory cytokines IL-17 and TNF-*α* decreased significantly in mice treated with smDCs alone or in combination with MTX when compared to levels in untreated CIA mouse sera (Figure 3(c)). The decrease in TNF-*α* was the most prominent in the 0.5 mg/kg MTX + smDCs combination group, and the serum level of IL-1*β* was significantly reduced when CIA mice were treated with MTX alone (Figure 3(c)). IL-1*β* reduction by MTX treatment was probably due to the MTX-mediated inhibition of IL-1*β* binding to its cell surface receptor.

### 3.4. Treg Increased and Th1/Th17 Decreased in CIA Mice after Treatment with smDCs Alone or in Combination with MTX

**Figure 4:**
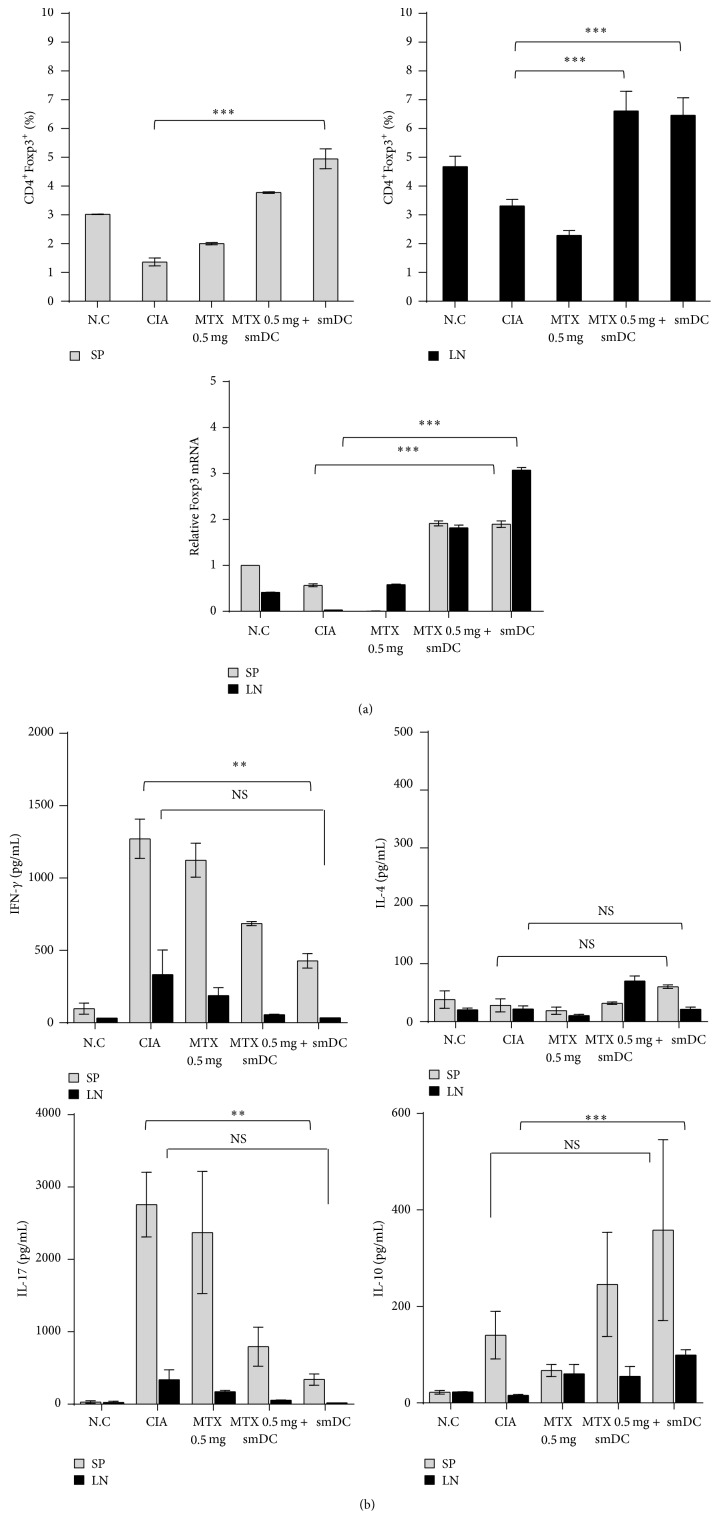
Combination therapy with smDCs and MTX enhanced Treg population and inhibited Th1/Th17 immunity with little influence on Th2 immunity. Advanced CIA mice were treated with smDCs, MTX, or smDCs and MTX, and T cell subsets were analyzed on day 77 after first CII injection. (a) Splenocytes and lymph nodes cells were cultured in the presence of CII (50 *μ*g/mL) for 72 h. Treg population and Foxp3 mRNA expression were analyzed by flow cytometry and qRT-PCR, respectively. (b) Single cell suspensions were cultured with 50 *μ*g/mL CII for 72 h. Cytokines secreted from the culture of splenocytes and lymph node cells were assessed. The levels of IFN-*γ*, IL-17, and IL-10 were measured by ELISA in the culture supernatants. Statistical data are shown from more than 3 different sets of experiments as mean ± SEM. ^*∗*^ *p* < 0.05, ^*∗∗*^ *p* < 0.001, ^*∗∗∗*^ *p* < 0.0001 in Student's *t*-test.

To examine immune status after smDC treatment, we assessed T cell subpopulations in the secondary lymphoid organs of treated mice. Splenic or lymph node (LN) T cells were restimulated with CII for 3 days and Treg/Th17 populations were measured by flow cytometric analysis after intracellular staining or by measuring cytokines in culture supernatants. Untreated CIA mice had reduced CD4^+^Foxp3^+^ Treg population in the spleen and LN compared to the normal control group. However, the Treg populations in both spleen and LN of CIA mice significantly increased after treatment with smDCs alone or in combination with MTX (Figure 4(a)). Increase of Treg population after the same treatments was further verified by examining Foxp3 mRNA level in CIA mice (Figure 4(a) lower panel). To evaluate the T cell-mediated immune deviation after these treatments, we examined the T cell subpopulations by measuring the concentration of Th1/Th2/Th17 cytokines in culture supernatants of T cells. The levels of interferon-*γ* (IFN-*γ*, Th1) and IL-17 (Th17) decreased significantly in both spleen and LN of CIA mice after treatment with smDCs alone or in combination with MTX, while IL-4 (Th2) was not significantly influenced by the same treatment (Figure 4(b)). In addition, different from IL-4, immune suppressive IL-10 increased significantly in the same treatment (Figure 4(b)). Our findings suggested that the preclinical outcomes shown in Figure 2 might be associated with the increase of Treg population and decrease of Th1/Th17 immunity after the therapy with smDCs alone or in combination with MTX. However, we cannot rule out the possibility that other immune responses might be involved in the preclinical outcomes after treatments.

### 3.5. Treatment of CIA Mice with smDCs or in Combination with MTX Induced Immune Suppressive Status in Secondary Lymphoid Organs

We examined the cytokine milieu and IDO expression in secondary lymphoid organs of CIA mice after treatment with smDCs alone or in combination with MTX. The mRNA levels of each cytokine and IDO were assessed from whole splenocytes and LN cells of CIA mice after therapy.

**Figure 5:**
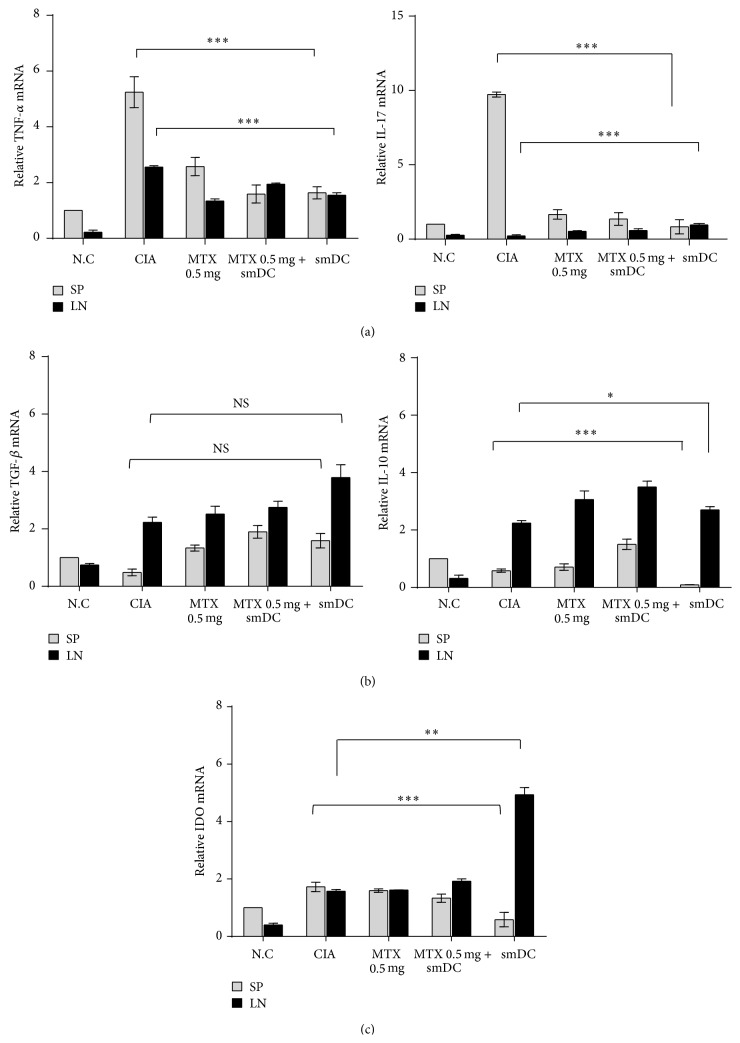
Treatment with smDCs or in combination with MTX made the secondary lymphoid organs of CIA mice become anti-inflammatory circumstances. Advanced CIA mice were treated with smDCs, MTX, or smDCs and MTX. Spleens and LNs were obtained from the treated mice on day 77 after first CII injection and total RNA was extracted from the organs. The mRNA levels of proinflammatory cytokines (a), anti-inflammatory cytokines (b), and immune-suppressive indoleamine 2,3 dioxygenase (IDO) (c) were analyzed by qRT-PCT using the ΔΔCT method and are expressed as a percent of control. Statistical data are shown from more than 3 different sets of experiments as mean ± SEM. ^*∗*^ *p* < 0.05, ^*∗∗*^ *p* < 0.001, ^*∗∗∗*^ *p* < 0.0001 in Student's *t*-test.

The mRNA levels of TNF-*α*, IFN-*γ*, and IL-17 were significantly reduced in the spleen of CIA mice after therapy (Figure 5(a)). The mRNA level of the immunosuppressive cytokine, TGF-*β*, significantly increased in the secondary lymphoid organs after treatment of CIA mice with smDCs alone while the level of anti-inflammatory cytokine IL-10 mRNA was little influenced by treatment (Figure 5(b)). It is well known that IDO is expressed in tolDCs and has a role in the induction of Treg cells [29–31]. The mRNA level of IDO was significantly enhanced only in the LN of CIA mice by therapy with smDCs alone, while the level was rather reduced in the spleen after the same treatment (Figure 5(c)). Combination (smDCs + MTX) therapy did not result in any significant change in IDO level in spleen or LN cells (Figure 5(c)). Even though treatment with MTX alone did not result in a clinical benefit to CIA mice (Figure 2), the MTX treatment reduced the level of proinflammatory cytokines TNF-*α*, IFN-*γ*, and IL-17 in the spleen (Figure 5(a)). It is worth noting that the reduction of proinflammatory cytokine levels after therapy was more dramatic in the spleen than in the LN, while the increase of immunosuppressive IDO and TGF-*β* was more significant in the LN than in the spleen (Figure 5).

### 3.6. smDCs Inhibited Th1 Proliferation and Induce Treg Population in Coculture with Splenic T Cells Obtained from CIA Mice

**Figure 6:**
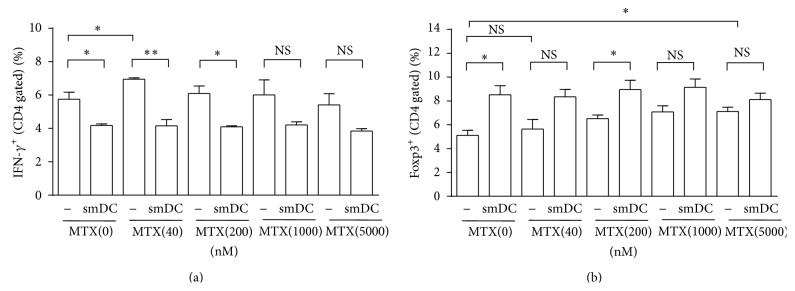
SmDC inhibited Th1 response and induced Treg response regardless of MTX in coculture with arthritogenic T cells. Arthritogenic T cells, collected from CIA mice 21 days after CII-inoculation, were cocultured with smDC in the presence or absence of MTX up to 5000 nM. (a) CD4^+^IFN-*γ* ^+^ Th1 cells were analyzed by FACS. (b) CD4^+^Foxp3^+^ Treg cells were analyzed by FACS. Statistical data are shown from 3 different mice as mean ± SEM. ^*∗*^ *p* < 0.05, ^*∗∗*^ *p* < 0.001 in Student's *t*-test.

We collected splenic T cells from CIA mice 21 days after CII-inoculation and cocultured them with smDCs in the presence or absence of MTX* in vitro*. IFN-*γ* ^+^ Th1 cell population significantly increased among the splenic T cells in CIA mice, whereas Th17 and IL-10^+^ T cells were still at a basal level at this time point in CII-inoculated mice. Coculture with smDCs decreased these IFN-*γ* ^+^ Th1 cell population in the splenic T cells regardless of the presence of MTX (Figure 6(a)), suggesting that smDCs were effective in inhibiting inflammatory Th1 cells but MTX was not, as was shown in* in vivo* data (Figure 4(b)). On the other hand, Foxp3^+^ Treg population increased in cocultures with smDCs or with MTX in a dose dependent manner (Figure 6(b)). However, smDCs and MTX did not show any synergistic effects on increasing of Treg populations (Figure 6(b)). These results were in good agreement with the* in vivo* clinical data (Figure 2) and the analysis of the splenocytes (Figure 4) of CIA mice after a combination therapy.

## 4. Discussion

The treatment of RA is based on MTX and other chemical DMARDs. Once DMARDs do not ameliorate symptoms, biologics, which usually target cytokines, are the next choice. However, biologics have severe side effects in some patients [32, 33] and the effect is diminished in some patients by anti-drug antibody formation [34–36]. Most importantly, biologics are not a fundamental treatment, since they generally reduce proinflammatory cytokines and do not remove a fundamental cause of the disease. Tolerogenic DCs (tolDCs) have been suggested as an attractive solution for fundamental treatment of autoimmune diseases [4, 37–39]. The possible mechanism of tolDC function in RA therapy is a reduction of Th17 population, induction of anergy and apoptosis in effector T cells, suppression of the activation of memory T cells, switching of Th1/Th2 balance to Th2 cells selectively, and induction of regulatory T cells (Treg) [40]. There are several ways to generate tolerogenic DCs [12]. NF-kB inhibitor treatment maintains DCs in an immature state and makes them tolerogenic [41]. Genetic modification of DCs becomes tolerogenic by expression of immunosuppressive genes (i.e., IL-4, IL-10, and CTLA-4) [42–46] or apoptosis-inducing factors (i.e., Fas, TRAIL) [47, 48] or inhibition of immunostimulatory molecules (i.e., CD80/CD86, IL-12) [49, 50]. Immunosuppressive cytokines IL-10/TGF-*β* [51–53] or rapamycin treatment [54], or short-term stimulation with LPS [28], has been used in efforts to make tolDCs. These tolDCs display an immature or semimature phenotype and reduced T cell stimulatory capacity.

TNF-mediated BMDCs are well known as semimature DCs (smDCs) for their surface phenotype, cytokine profile, and biological characteristics in comparison with imDCs and mDCs [55, 56]. We reported that CII-loaded smDCs have a prophylactic effect in CIA mice and the preventative effect is dependent on the tolerogenic functions of low-dose smDCs [14]. In the present study, we demonstrate that TNF-*α*-derived smDCs also have therapeutic potential in advanced CIA mice with arthritic scores of 2~3, and this therapeutic function was still effective when combined with MTX, a first-line agent for RA treatment. The CIA mice treated with CII-smDCs alone or in combination with MTX had a significant reduction in CII-specific cellular and humoral immunities together with proinflammatory cytokines. Foxp3 Treg populations were markedly enhanced while Th1 and Th17 populations decreased significantly with little effect on Th2 immunity. These treatments also increased IL-10-expressing T cells in the spleen and induced an anti-inflammatory state in the secondary lymphoid organs of CIA mice. These data suggest that the treatment with CII-smDCs alone or in combination with MTX is effective in controlling disease progression in advanced CIA in mice by shifting inflammatory immune environments to a relative anti-inflammatory state.

Even though MTX alone did produce a clear clinical benefit in advanced CIA, treatment of early onset of CIA with MTX alone slightly delayed CIA progression, probably due to MTX-mediated inhibition of proinflammatory cytokine expression in the spleen. In the mice that received combination therapy with smDCs and MTX, the optimal concentration of MTX was 0.5 mg/kg, and low (0.1 mg/kg) and high (1.0 mg/kg) doses of MTX impaired the smDCs-mediated therapeutic efficacy rather than inducing a synergistic effect. Therefore, special attention should be paid to the optimal concentration of MTX in combination therapy with smDCs.

MTX, a standard therapy in RA, is known to affect the immune system [16–19]. To use smDCs in clinic to treat RA in conjunction with MTX, we have to prove that combination therapy with smDCs and MTX is more effective than MTX alone. The MTX-only regimen did not have any therapeutic effect in advanced CIA. However, the combination of MTX and smDCs was as effective as smDCs alone in the treatment of advanced CIA. Thus, MTX did not damage the tolerogenic function of smDCs. Our findings suggest that smDCs can be used to treat patients with inadequate response to MTX.

## 5. Conclusion

Treatment of CIA mice with smDCs alone or in combination with MTX was effective in controlling arthritis by decreasing the population of CII-specific Th17 and Th1 cells in the spleen and increasing the population of Treg cells dominant in the LN, leading to inhibition of disease progression.

## Figures and Tables

**Table 1:** Sequences of primers for RT-PCR.

	Primer sequence	
TNF-*α*	Forward	ACC ACT CTC CCT TTG CAG AAC TCA
	Reverse	TCT CAT GCA CCA TCA AGG ACT

IL-17	Forward	TCC ACC GCA ATG AAG ACC CTG ATA
	Reverse	ACC AGC ATC TTC TCG ACC CTG AAA

TGF-*β*	Forward	GTG CGG CAG CTG TAC ATT GAC TTT
	Reverse	TGT ACT GTG TGT CCA GGC TCC AAA

IL-10	Forward	GCT CTT GCA CTA CCA AAG CCA CAA
	Reverse	AGT AAG AGC AGG CAG CAT AGC AGT

IDO	Forward	TCG GAA GAG CCC TCA AAT GTG GAA
	Reverse	TGG AGC TTG CTA CAC TAA GGC CAA

## Abbreviations

DC: Dendritic cells
tDC: Tolerogenic dendritic cells
smDC: Semimature dendritic cells
RA: Rheumatoid arthritis
MTX: Methotrexate
CIA: Collagen-induced arthritis.
